# Correlation between Humphrey Field Analyser perimetric outcomes and macular thickness in diabetic retinopathy patients, diabetic individuals without retinopathy, and control subjects in Trinidad and Tobago

**DOI:** 10.1186/s13104-026-07638-4

**Published:** 2026-01-18

**Authors:** Kingsley Ekemiri, Chidum Ezenwaka, Reyanna Ramkissoon, Anil Ramsaran, Chioma Ekemiri, Esther Daniel, Kathy-Ann Lootawan, Linda Ekwe

**Affiliations:** 1 School of Optometry, Faculty of Health and Medical Sciences, Abia State University, Uturu, Nigeria; 2 Optometry Unit, Department of Clinical Surgical Sciences, Faculty of Medical Sciences, The University of the West Indies, St. Augustine, Trinidad and Tobago; 3 Department of Optometry, Faculty of Health Sciences, University of Kwazulu-Natal, Kwazulu-Natal City, South Africa; 4 Department of Para-Clinical Sciences, Faculty of Medical Sciences, The University of the West Indies, St Augustine Campus, St Augustine, Trinidad and Tobago; 5 Department of Health Promotion, The University of the West Indies, St. Augustine Campus, St Augustine, Trinidad and Tobago; 6 School of Nursing, Faculty of Medical Sciences, The University of the West Indies, St. Augustine, St Augustine, Trinidad and Tobago; 7 Optometry Department, Federal Medical Center, Makurdi, Nigeria

**Keywords:** Optical coherence tomography, Retinopathy, Diabetics, Macula, Visual field assessment

## Abstract

**Objective:**

This study examined the relationship between visual field performance measured with the Humphrey Field Analyzer and macular thickness measured using optical coherence tomography across three groups: individuals without diabetes, individuals with diabetes but no diabetic retinopathy, and individuals with diabetic retinopathy. The aim was to determine whether structural and functional measures show meaningful associations within and across disease stages in a Trinidad and Tobago population.

**Results:**

In unadjusted comparisons, macular and visual-field measures differed across groups (all *p* < 0.001). After age adjustment, most between-group differences were no longer significant, and age independently predicted VFI, confirming age as a major confounder. Within-group, age-adjusted associations persisted in DR (OD): lower VFI correlated with greater superior (partial *r* = − 0.519, *p* = 0.027) and temporal ETDRS thickness (partial *r* = − 0.585, *p* = 0.011), and higher PSD correlated with greater superior (partial r = + 0.603, *p* = 0.013) and temporal thickness (partial r = + 0.592, *p* = 0.016); all four remained significant after FDR correction. Macular total volume showed limited functional relevance. In DM_no_DR (exploratory), small-sample regressions suggested similar direction, but estimates were imprecise. MD was not analyzed via ANCOVA due to incomplete data.

**Supplementary Information:**

The online version contains supplementary material available at 10.1186/s13104-026-07638-4.

## Introduction

Diabetic retinopathy (DR) is a significant global health issue, particularly in populations with a high prevalence of diabetes mellitus (DM) such as Trinidad and Tobago [1]. With the advancement of technology, the integration of various diagnostic modalities has enabled a more comprehensive understanding of the pathophysiology and progression of diabetic retinopathy. Among these, the Humphrey Field Analyser (HFA) provides quantitative assessment of visual field defects, reflecting functional impairment, while optical coherence tomography (OCT) allows for precise evaluation of macular thickness and retinal layers, thereby identifying structural alterations associated with diabetic macular edema (DME) and other retinal complications [2–5].

Studying these functional and structural aspects in combination is important because early functional loss has been suggested to occur before visible retinopathy, while structural changes can predict subsequent decline in visual performance. Together, these techniques enhance the diagnosis, management, and treatment of diabetic eye diseases, ultimately improving patient outcomes. The clinical value of these modalities has been extensively demonstrated: HFA is widely used to detect visual field loss in glaucoma and other ocular diseases [6], and OCT is particularly important for evaluating macular thickness, detecting DME, and classifying its morphological patterns [7–9]. The importance of OCT in detecting and managing DME, specifically identifying five morphologic patterns of DME using OCT, were investigated [8]. OCT findings have also been emphasized as useful in diagnosing and monitoring retinal diseases despite known limitations [9].

Evidence further shows that macular thickness correlates with glycemic control and disease duration, even in the absence of clinically detectable DME [10]. The potential of ultra-widefield imaging to identify quantitative metrics for classification and prognostication of DR has also been explored [10]. Additionally, while the presence of DR has been associated with systemic neurodegeneration, its severity does not appear to influence this association [11]. Reviews have also underscored the role of OCT and fluorescein angiography in assessing DR and guiding clinical decisions [12, 13]. These studies collectively highlight the need for further research that examines the relationship between visual field parameters (functional changes) and macular thickness measurements (structural changes) across different stages of diabetic retinopathy.

Although OCT-derived macular thickness primarily reflects central retinal structure, the macula particularly the ganglion cell and inner plexiform layer plays a crucial role in overall visual field sensitivity and retinal neural processing [16, 19]. Standard automated perimetry protocols such as the 24 − 2 and 30 − 2 strategies, which were employed in this study, include paracentral test points that anatomically correspond to macular and parafoveal regions rather than purely peripheral locations [6, 18]. Some studies suggest that early neurodegenerative changes in diabetes may originate in the macula even before clinically detectable retinopathy and subsequently influence broader retinal function [10, 14], which may influence HFA outcomes to diffuse or generalized visual field defects through transsynaptic damage and widespread retinal dysfunction [11, 15].

Therefore, correlating macular structure with HFA outcomes may provide insight into possible early subclinical dysfunction [17, 18]. Nevertheless, future studies incorporating central visual field protocols such as the 10 − 2 strategy may offer even greater precision in evaluating macula-specific functional loss [19]. Moreover, there is a paucity of such investigations within specific ethnic populations, such as those in Trinidad and Tobago, where unique genetic and environmental factors may influence disease patterns. In this study, we aim to bridge this gap by exploring the correlation between HFA perimetry outcomes and OCT-derived macular thickness in diabetic retinopathy patients, diabetic individuals without retinopathy, and control subjects from Trinidad and Tobago. By explicitly examining the interplay between functional visual field parameters and structural macular changes, this study aims to explore whether structural and functional measures show meaningful associations across disease stages. This research may also provide insights into how functional deficits and structural retinal alterations evolve together in DR, with implications for early intervention.

## Methods

### Study design and setting

This cross-sectional study was conducted at the University Optometry Clinic in Trinidad and Tobago between January 1 and March 30, 2023. The clinic, established in 2009, provides specialized services in primary eye care, low vision, binocular vision, and medical optometry.

### Participants and sampling

A total of 104 adults attending the clinic during the study period were recruited using convenience sampling and assigned to one of three groups:

1. Control group: 70 individuals without diabetes.
2. DM without diabetic retinopathy: 16 individuals.
3. DM with non-proliferative or proliferative diabetic retinopathy: 18 individuals.

Eligibility criteria included adults aged ≥ 18 years who were able to provide informed consent and complete all visual field and OCT procedures. Exclusion criteria were: Critically ill individuals, Cognitive or communication impairment, Inability to perform reliable automated perimetry and Clinically Significant Diabetic Macular Edema (CSDME) or OCT-confirmed macular edema, to avoid confounding macular thickness measurements. Demographic and knowledge–attitude–practice (KAP) data were collected for clinical context but were not included in this analysis, which focused exclusively on structural and functional ocular outcomes.

### Clinical examination and data collection

Participants were contacted by telephone and invited for assessment. Upon arrival, capillary HbA1c was measured using the A1CNow + system (PTS Diagnostics) to confirm glycemic status, with ≥ 6.5% used to classify diabetes in accordance with NHANES criteria. Each participant underwent a standardized examination protocol that included: Best-corrected visual acuity using an ETDRS chart, Contrast sensitivity using the Mars Contrast Sensitivity Test, Intraocular pressure measured with a Zeiss AT030 Goldmann applanation tonometer.

### Visual field assessment

Functional evaluation of the visual pathways was performed using the Humphrey Field Analyzer 3 (Zeiss HFA 3/860) with the 24 − 2 SITA-Standard protocol, which provides a quantitative assessment of central and paracentral visual sensitivity. Standard reliability indices fixation losses, false-positive responses, and false-negative responses were reviewed for each examination, and unreliable fields were excluded.

From each reliable visual field test, the following functional parameters were extracted: Visual Field Index (VFI), Pattern Standard Deviation (PSD), Mean Deviation (MD). These indices provided an overview of global visual function, localized defects, and overall sensitivity loss.

### Optical coherence tomography (OCT)

After pharmacologic dilation with 1% tropicamide, macular imaging was performed using the Cirrus 6000 OCT system (Carl Zeiss Meditec). A high-resolution macular cube scan was captured for each study eye to quantify retinal thickness and structural integrity. Structural parameters derived included: Macular average thickness, Macular center thickness, Total macular volume and ETDRS quadrant thicknesses (superior, temporal, nasal, inferior). All OCT scans were independently reviewed by two trained graders to ensure adequate quality. Segmentation errors were corrected manually when present. A circumpapillary retinal nerve fiber layer (cp-RNFL) scan was also obtained; however, these data were not included in the primary analysis.

### Grading of diabetic retinopathy

The severity of diabetic retinopathy was determined using fundus photographs and corresponding OCT findings, following the International Clinical Diabetic Retinopathy (ICDR) Severity Scale. Each eye was classified as:

- No diabetic retinopathy (No DR).
- Mild or moderate non-proliferative diabetic retinopathy (NPDR).
- Severe NPDR.
- Proliferative diabetic retinopathy (PDR)

This grading ensured consistent categorization of participants based on structural disease severity.

### Outcome measures

The study examined both structural and functional indicators of retinal health.

*Functional outcomes (HFA)* Visual Field Index (VFI), Pattern Standard Deviation (PSD), Mean Deviation (MD).

*Structural outcomes (OCT)* Macular average thickness, Macular center thickness, Total macular volume, ETDRS quadrant thicknesses.

These combined metrics enabled an integrated assessment of retinal structure–function relationships across varying stages of diabetes and diabetic retinopathy.

### Data processing and analysis

Quantitative variables were summarized as mean ± standard deviation (SD). Initial group comparisons used independent t-tests for continuous variables and Pearson’s Chi-square tests for categorical variables. One-way analysis of variance (ANOVA) with Bonferroni post hoc adjustments was used to explore preliminary differences across the three study groups. Pearson correlation coefficients assessed associations between structural and functional parameters.

Because the control group was substantially younger than both diabetic groups, age was identified as a major confounding factor. To address this imbalance, Analysis of Covariance (ANCOVA) was conducted for each primary outcome, with Group as the fixed factor and Age as the covariate. Separate ANCOVA models were run for: VFI, PSD, Macular average thickness, Macular center thickness, Macular total volume, ETDRS quadrant thicknesses (superior, temporal, nasal, inferior). Mean Deviation (MD) was not included in ANCOVA due to incomplete data across groups.A p-value < 0.05 was considered statistically significant. All analyses were conducted using IBM SPSS Statistics for Windows, Version 29.0 (IBM Corp., Armonk, NY, USA).

### Ethics approval and consent to participate

All procedures involving human participants were conducted in full accordance with the ethical standards of the institutional research committee and with the principles outlined in the Declaration of Helsinki and its later amendments. Ethical approval was granted by the University of the West Indies, St. Augustine Campus Ethics Committee (Approval No. *CREC-SA 1805/10/2022*), and written informed consent was obtained from all participants prior to their inclusion in the study.

## Results

### Characteristics of the study participants

A total of 104 participants were enrolled in the study, including 70 non-diabetic controls (67.3%), 16 individuals with diabetes but no diabetic retinopathy (15.4%), and 18 individuals with diabetic retinopathy (17.3%). The mean age differed substantially across groups, increasing progressively with disease severity.

Controls were the youngest group (25.9 ± 7.35 years), followed by diabetics without retinopathy (52.0 ± 12.51 years) and participants with diabetic retinopathy (61.7 ± 8.98 years).

Both sexes were represented in all groups, with a slightly higher proportion of females overall. Participants resided in both urban and rural areas; however, diabetic retinopathy was more common among rural residents compared to controls. (Table 1).

**Table 1:** Demographic characteristics of control participants, diabetics without retinopathy, and diabetics with retinopathy (Trinidad and Tobago, 2023)

Variable	Control (*n* = 70)	DM without DR (*n* = 16)	DR (*n* = 18)
Sex			
Male	21 (30.0%)	2 (12.5%)	10 (55.6%)
Female	49 (70.0%)	14 (87.5%)	8 (44.4%)
Residence			
Urban	42 (60.0%)	10 (62.5%)	10 (55.6%)
Rural	28 (40.0%)	6 (37.5%)	8 (44.4%)
Mean age (years ± SD)	25.9 ± 7.35	52.0 ± 12.51	61.7 ± 8.98
Number of eyes examined	140 (67.3%)	32 (15.4%)	36 (17.3%)

### Clinical characteristics of study participants

Significant functional and structural differences were observed across the three groups (controls, diabetics without retinopathy, and diabetics with retinopathy). Visual field performance declined progressively with disease severity. The Visual Field Index (VFI) was highest in controls (OD: 98.84 ± 1.15%), decreased in diabetics without retinopathy (86.50 ± 27.41%), and was lowest in those with diabetic retinopathy (88.00 ± 23.52%) (*p* < 0.001). Pattern Standard Deviation (PSD) increased from 1.61 ± 0.32 dB in controls to 2.77 ± 2.42 dB in diabetics without retinopathy and 3.23 ± 2.50 dB in the retinopathy group (*p* < 0.001), indicating greater visual field irregularity. Similarly, mean deviation (MD) became more negative across groups, reflecting worsening global visual field sensitivity.

### Age-adjusted structure–function coupling in diabetic eyes (OD/OS)

After adjusting for age, regional macular thickening showed clear functional correlates in diabetic eyes. In OD, superior and temporal ETDRS thicknesses were positively associated with PSD (partial *r* = 0.603, *p* = 0.010; partial *r* = 0.592, *p* = 0.012, respectively) and negatively associated with VFI (partial *r* = − 0.519, *p* = 0.023; partial *r* = − 0.585, *p* = 0.008). In OS, superior ETDRS thickness correlated negatively with VFI (partial *r* = − 0.648, *p* = 0.003). Optic-disc metrics in OS also tracked function: PSD increased with rim area (partial *r* = 0.644, *p* = 0.005) and decreased with cup area (partial *r* = − 0.694, *p* = 0.002). Corresponding Pearson (unadjusted) correlations were generally weaker and often non-significant, consistent with age confounding; therefore, the age-adjusted effects provide the most clinically informative estimates. These age-adjusted and unadjusted associations are summarized in Table 2.

**Table 2:** Structure–function associations in diabetic participants (OD/OS): pearson vs. age-adjusted partial correlations

Eye	Visual field index	Structural parameter	Partial *r*	*p*-value
OD (macula)	PSD	ETDRS superior	**+ 0.603**	**0.010**
OD (macula)	PSD	ETDRS temporal	**+ 0.592**	**0.012**
OD (macula)	VFI	ETDRS superior	**−0.519**	**0.023**
OD (macula)	VFI	ETDRS temporal	**−0.585**	**0.008**
OS (macula)	VFI	ETDRS superior	**−0.648**	**0.003**
OS (disc)	PSD	Rim area	**+ 0.644**	**0.005**
OS (disc)	PSD	Cup area	**−0.694**	**0.002**
OS (disc)	MD	Disc area	**−0.515**	**0.024**

Bold type denotes statistically significant results (*p* < 0.05) based on age-adjusted partial correlation analysis

*OD/OS* right/left eye, *VFI* Visual Field Index, *PSD* Pattern Standard Deviation, *MD* Mean Deviation, *ETDRS* macular sector thickness from OCT, disc metrics from ONH OCT

### Analysis of macular thickness (ETDRS) versus visual-field outcomes in diabetics without retinopathy (DM_no_DR, OD)

Using age-adjusted linear models (covariate: Age), temporal and superior macular sector thicknesses were independently associated with visual-field performance (Table 3). For VFI, both ETDRS temporal (β_std = − 0.57, 95% CI for unstandardized β − 0.00095 to − 0.00015, *p* = 0.011) and ETDRS superior thickness (β_std = − 0.58, 95% CI − 0.00120 to − 0.00008, *p* = 0.027) showed moderate negative associations, indicating that thicker sectors were linked to lower global field indices after accounting for age. Each ETDRS term explained substantial additional variance beyond age (ΔR² = 0.33 for temporal; ΔR² = 0.26 for superior; R²_full = 0.38 and 0.31, respectively).

**Table 3:** Age-adjusted regression models in DM_no_DR (OD only): ETDRS predictors of visual-field indices

Visual field	Predictor (ETDRS)	*n*	β_std_struct	β_struct 95% CI	p_struct	*R*²_age_only	*R*²_full (Age + ETDRS)	ΔR²_struct
VFI_OD	ETDRS_Temp_OD	19	−0.57	−0.00095 to − 0.00015	0.011	0.052	0.376	0.325
VFI_OD	ETDRS_Sup_OD	19	−0.58	−0.00120 to − 0.00008	0.027	0.052	0.307	0.255
PSD_OD	ETDRS_Sup_OD	17	+ 0.73	+ 0.0039 to + 0.0285	0.013	0.006	0.368	0.361

*N*OD right eye, *ETDRS* macular sector thickness, *VFI* Visual Field Index, *PSD* Pattern Standard Deviation. Models adjust for Age; ΔR²_struct denotes variance explained by the ETDRS term beyond Age. Results are exploratory due to small sample size

For PSD, ETDRS superior thickness was positively associated with localized field irregularity (β_std = + 0.73, 95% CI + 0.0039 to + 0.0285, *p* = 0.013), contributing an additional ΔR² = 0.36 beyond the age-only model (R²_full = 0.37). These effects are directionally consistent with the age-adjusted partial-correlation findings in the broader diabetic cohort (superior/temporal sectors ↔ VFI↓, PSD↑).

### Age-adjusted structure–function relationships in the DR subgroup (OD)

In eyes with diabetic retinopathy, age-adjusted partial correlations showed that lower VFI was associated with greater superior (partial *r* = − 0.519, *p* = 0.027; *n* = 19) and temporal macular thickness (partial *r* = − 0.585, *p* = 0.011; *n* = 19). Conversely, higher PSD related to greater superior (partial r = + 0.603, *p* = 0.013; *n* = 17) and temporal thickness (partial r = + 0.592, *p* = 0.016; *n* = 17). All four associations remained significant after FDR correction across the tested pairs. Structural-coherence analyses indicated that macular total volume showed only modest, non-significant associations with ETDRS sector thickness (*r* ≈ − 0.09 to − 0.42; all *p* > 0.07). Taken together, these findings indicate that, in DR, superior and temporal macular sectors retain a measurable, age-adjusted coupling with both global (VFI) and localized (PSD) visual-field deficits (Table 4).

**Table 4:** Pearson (unadjusted) and age-adjusted partial correlations between Humphrey Field Analyzer visual-field indices and ETDRS macular sector thickness in eyes with diabetic retinopathy (OD). Pearson *r* values represent unadjusted correlations, while partial *r* values are adjusted for age. VFI shows negative associations and PSD positive associations with superior and temporal ETDRS macular thickness

VF index	Macular parameter	*n*	Pearson *r*	Pearson *p*	Partial *r* (age-adjusted)	Partial *p*	FDR (q < 0.05)
VFI_OD	ETDRS_Sup_OD	19	−0.3255	0.1739	**−0.5190**	**0.0273**	Yes
VFI_OD	ETDRS_Temp_OD	19	−0.5572	**0.0132**	**−0.5851**	**0.0107**	Yes
PSD_OD	ETDRS_Sup_OD	17	0.4485	0.0710	**0.6031**	**0.0134**	Yes
PSD_OD	ETDRS_Temp_OD	17	**0.5838**	**0.0139**	**0.5923**	**0.0156**	Yes

Bold indicates significance (*p* < 0.05; FDR *q* < 0.05)

*Pearson* *r* unadjusted; *partial* *r* age-adjusted correlations*; VFI* Visual Field Index; *PSD* Pattern Standard Deviation; *ETDRS* Early Treatment Diabetic Retinopathy Study; *OD* right eye

## Discussion

This study evaluates retinal structure–function relationships across controls, DM_no_DR, and DR using OCT and automated perimetry. In unadjusted analyses, visual-field indices (VFI, PSD, MD) and macular metrics (thickness, volume, ETDRS sectors) appeared progressively abnormal from controls to DR. After adjusting for age, however, most group differences attenuated and were no longer significant (Table 5), confirming age as a major confounder. Within this constraint, age-adjusted association analyses demonstrated biologically plausible, sector-specific coupling between macular structure and visual-field function, particularly in the superior and temporal macular regions.

**Table 5:** Comparison of functional visual field and macular structural parameters among controls, diabetics without retinopathy, and diabetics with retinopathy, including age-adjusted ANCOVA p-values

Outcome measure	Controls (*n* = 70)	DM without DR (*n* = 16)	DR (*n* = 18)	Unadjusted *p* (ANOVA)	Age-adjusted *p* (ANCOVA)
Visual field parameters (OD)					
VFI (%)	98.84 ± 1.15	86.50 ± 27.41	88.00 ± 23.52	< 0.001	0.94
PSD (dB)	1.61 ± 0.32	2.77 ± 2.42	3.23 ± 2.50	< 0.001	0.12
Macular thickness measures (OD)					
Average thickness (µm)	264.81 ± 11.12	263.96 ± 10.26	260.94 ± 35.48	< 0.001	0.11
Centre thickness (µm)	181.45 ± 28.01	173.87 ± 13.75	233.00 ± 107.70	< 0.001	0.25
Total macular volume (mm³)	7.44 ± 0.32	7.45 ± 0.26	7.41 ± 0.98	< 0.001	0.82
ETDRS quadrants (OD, µm)					
Superior	261.11 ± 12.00	258.75 ± 8.57	255.00 ± 15.45	< 0.001	0.11
Temporal	250.10 ± 13.26	247.87 ± 9.40	253.30 ± 30.04	< 0.001	0.15
Nasal	274.01 ± 12.16	271.75 ± 10.40	271.55 ± 30.29	< 0.001	0.87
Inferior	257.85 ± 16.50	254.87 ± 16.45	257.66 ± 32.64	< 0.001	0.14

*DMNRD* diabetes mellitus without diabetic retinopathy, *DR* diabetic retinopathy, *OD* right eye, *SD* standard deviation, *VFI* Visual Field Index, *PSD* Pattern Standard Deviation, *MD* Mean Deviation. Values are presented as mean ± SD. Unadjusted p-values were obtained using one-way ANOVA. Age-adjusted p-values were obtained using ANCOVA with age included as a covariate. MD values were not included in ANCOVA due to insufficient complete data across groups

DM_no_DR showed trends toward subtle macular alterations and functional deficits (lower VFI, higher PSD). Although age adjustment weakened between-group contrasts, age-adjusted within diabetic analyses suggested that superior and temporal macular thickness relates to both VFI and PSD, consistent with the concept that neurodegeneration may precede clinically visible microvascular change [10, 13, 14]. Given the small sample size and multiple testing considerations, these signals should be viewed as preliminary and require confirmation in larger, age-balanced cohorts.

In DR eyes, macular structure was more diffusely altered, with strong internal coherence across ETDRS sectors. Importantly, after age adjustment, lower VFI was associated with thicker superior and temporal sectors, and higher PSD showed the same sectoral pattern; all associations remained significant after FDR correction (Table 2). These findings align with reports that progressive retinopathy is accompanied by inner-retinal thinning or reshaping, ganglion-cell complex disturbance, and functional impairment [21–24]. They also underscore that sectoral OCT metrics rather than global macular volume may offer greater sensitivity in reflecting functional loss.

Our sector-specific findings are directionally consistent with prior evidence that macular structural integrity particularly within the ganglion-cell and inner-plexiform layers tracks visual-field sensitivity [16–19]. In diabetes, macular thinning and dysfunction have been linked to disease duration, hyperglycemia, and inflammatory mediators (e.g., VEGF, MCP-1), supporting a neuroinflammatory–ischemic pathway that may occur even before ophthalmoscopic retinopathy is evident [10, 14, 20]. In DR and DME, edema burden and photoreceptor disruption further correlate with visual impairment and disease severity [21–24]. The present data extend this literature by demonstrating that, even after accounting for age, superior and temporal macular sectors provide the clearest functional correlates, whereas global macular volume contributes comparatively little additional information.

### Clinical implications

1. Screening and monitoring: Sectoral OCT measurements—particularly superior and temporal ETDRS regions may complement automated perimetry in identifying eyes at functional risk, offering additional insight beyond visual acuity alone.
2. Integrated assessment: Combining OCT with automated perimetry provides a more comprehensive evaluation of retinal status than either modality alone, especially in the context of diabetic retinopathy.
3. Risk stratification: Eyes demonstrating concordant sectoral OCT changes and visual-field abnormalities may warrant closer follow-up and patient counseling, pending validation in larger, age-balanced samples.

### Limitations and next steps

The principal limitations of this study include age imbalance across groups, small diabetic subgroups (and missing MD data), and potential heterogeneity introduced by DME, which can produce a mixture of macular thickening and thinning. Future studies should aim to recruit age-balanced cohorts, exclude or stratify participants by DME status or explicitly model its effects and incorporate central visual-field protocols (10–2) to improve macular specificity. Longitudinal designs will be essential to determine whether sectoral OCT abnormalities predict subsequent visual field decline beyond age and glycemic control.

## Conclusion

This study suggests that diabetic eye disease is characterized by both neuroretinal dysfunction and structural alteration across the diabetic spectrum. However, after adjusting for age, most crude between-group differences were markedly reduced, underscoring age as a major confounder. Within this context, sector-specific, age-adjusted structure–function relationships were observed, particularly in the superior and temporal macular regions of eyes with diabetic retinopathy, where greater macular thickness corresponded to lower VFI and higher PSD. In diabetics without retinopathy, similar associations were directionally consistent but remain exploratory due to the small sample size.

These findings support the clinical value of integrating OCT especially sectoral ETDRS analysis with automated perimetry to enhance early detection, risk stratification, and monitoring, while also highlighting the importance of interpreting unadjusted comparisons with caution. Future research should adopt longitudinal, age-balanced designs; explicitly exclude or stratify by DME; incorporate central visual-field protocols (e.g., 10 − 2); and evaluate whether early macular sector changes predict subsequent functional decline or respond to neuroprotective or anti-inflammatory therapies.

## Supplementary Information

Below is the link to the electronic supplementary material.

Supplementary Material 1.

## Data Availability

All data supporting the findings of this study are included within the manuscript and its supplementary information files.
