# Supplementary material for: Correlation between Humphrey Field Analyser perimetric outcomes and macular thickness in diabetic retinopathy patients, diabetic individuals without retinopathy, and control subjects in Trinidad and Tobago
Source: BMC Res Notes. 2026 Jan 18;19:58. doi: 10.1186/s13104-026-07638-4 (PMC12895719; doi:10.1186/s13104-026-07638-4)
Supplement: Supplementary file 1 — Supplementary Material 1. [file 13104_2026_7638_MOESM1_ESM.docx]

**Questionnaire:**

**Section 1: Demographic data. Gathering of general information of the participants. (Name, age, Level of education currently pursuing, class)**

**First name Middle name Last name**

**________________ _________________ ___________________**

**Date:**

**_______________**

**Contact number:**

**___________________**

**Email Address:**

**________________________**

**Date of Birth: dd/mm/yy**

**________________**

**How old are you?**

**_______________**

**Gender:**

**Male 󠆹󠄀**

**Female󠄀 󠄀󠄀**

**Other󠄀 󠄀󠄀**

**What is your current Occupation/Task?**

**_____________________________**

**What is the Level of education that you are currently pursing?**

**None 󠄀󠆹 󠄀**

**Primary 󠄀 󠄀󠆹 󠄀**

**Secondary 󠄀󠄀**

**Territory 󠄀󠆹**

**Section 2: General knowledge and perception on diabetes and diabetic Mellitus.**

**Do you know what is Diabetes Mellitus?**

**Yes󠆹󠄀 No󠆹󠄀**

**What would you rate your level of understanding of diabetes mellitus?**

**Very low󠆹󠄀 Low 󠆹󠄀Average󠆹󠄀 High 󠆹󠄀Very High 󠆹󠄀**

**When last have you visited your Primary Care Doctor?**

**______________________**

**When was your Last Eye Examination?**

**______________________________________**

**Where was your Last Eye Examination?**

**_______________________________________**

**Section 3: Awareness and Attitudes towards individuals and/or themselves who have diabetes or diabetic mellitus**

**Do you know any family members that has Diabetes Mellitus?**

**Yes󠆹󠄀 No󠆹󠄀**

**if Yes, states how many**

**________________________**

**If yes, how has diabetes affected your life**

**____________________________________________________________**

**Very low󠆹󠄀 Low 󠆹󠄀Average󠆹󠄀 High 󠆹󠄀Very High 󠆹󠄀**

**Do you have Diabetes Mellitus?**

**Yes󠆹󠄀 No󠆹󠄀 Not sure 󠆹󠄀**

**Section 4: The audience’s general practices that they exhibit and follow to prevent and/or treat diabetes or diabetic mellitus**

**If yes to diabetes, how do you manage it?**

**__________________________________________**

**If yes to family having diabetes, how do they manage it?**

**_________________________________________________**

**What do you do to prevent yourself from becoming diabetic?**

**__________________________________________________________________**

**What do you do to treat yourself or family members that are diabetes?**

**__________________________________________________________________**
